# Role of epidermal growth factor receptor inhibitor-induced interferon pathway signaling in the head and neck squamous cell carcinoma therapeutic response

**DOI:** 10.1186/s12967-021-02706-8

**Published:** 2021-01-23

**Authors:** Sean P. Korpela, Trista K. Hinz, Ayman Oweida, Jihye Kim, Jacob Calhoun, Robert Ferris, Raphael A. Nemenoff, Sana D. Karam, Eric T. Clambey, Lynn E. Heasley

**Affiliations:** 1 Department of Craniofacial Biology, School of Dental Medicine, University of Colorado Anschutz Medical Campus, 12801 E. 17th Ave, Aurora, CO 80045 USA; 2 Department of Medicine, University of Colorado Anschutz Medical Campus, Aurora, CO USA; 3 Department of Radiation Oncology, University of Colorado Anschutz Medical Campus, Aurora, CO USA; 4 Department of Anesthesiology, University of Colorado Anschutz Medical Campus, Aurora, CO USA; 5 Department of Nuclear Medicine and Radiobiology, Universite de Sherbrooke, Sherbrooke, Québec Canada; 6 Eastern Colorado VA Healthcare System, Rocky Mountain Regional VA Medical Center, Aurora, CO USA; 7 Departments of Otolaryngology and Immunology, University of Pittsburgh, Pittsburgh, PA USA

**Keywords:** EGFR, HNSCC, Tyrosine kinase inhibitor, Transcriptional reprogramming, Interferon response

## Abstract

**Background:**

Epidermal growth factor receptor (EGFR) is frequently amplified or overexpressed in head and neck squamous cell carcinoma (HNSCC) and is a clinically validated target for the therapeutic antibody, cetuximab, in the management of this cancer. The degree of response to EGFR inhibitors measured by tumor shrinkage varies widely among HNSCC patients, and the biological mechanisms that underlie therapeutic heterogeneity amongst HNSCC patients remain ill-defined.

**Methods:**

EGFR-dependent human and murine HNSCC cell lines were treated with the EGFR/ERBB inhibitors, gefitinib and AZD8931, and submitted to RNAseq, GSEA, and qRT-PCR. Conditioned media was analyzed by ELISA and Luminex assays. Murine HNSCC tumors were stained for T cell markers by immunofluorescence. Primary HSNCC patient specimens treated with single agent cetuximab were stained with Vectra multispectral immunofluorescence.

**Results:**

The transcriptional reprogramming response to EGFR/ERBB-specific TKIs was measured in a panel of EGFR-dependent human HNSCC cell lines and interferon (IFN) α and γ responses identified as top-ranked TKI-induced pathways. Despite similar drug sensitivity, responses among 7 cell lines varied quantitatively and qualitatively, especially regarding the induced chemokine and cytokine profiles. Of note, the anti-tumorigenic chemokine, CXCL10, and the pro-tumorigenic factor, IL6, exhibited wide-ranging and non-overlapping induction. Similarly, AZD8931 exerted potent growth inhibition, IFNα/IFNγ pathway activation, and CXCL10 induction in murine B4B8 HNSCC cells. AZD8931 treatment of immune-competent mice bearing orthotopic B4B8 tumors increased CD8 + T cell content and the therapeutic response was abrogated in nu/nu mice relative to BALB/c mice. Finally, Vectra 3.0 analysis of HNSCC patient tumors prior to and after 3–4 weeks of single agent cetuximab treatment revealed increased CD8 + T cell content in specimens from patients exhibiting a therapeutic response relative to non-responders.

**Conclusions:**

The findings reveal heterogeneous, tumor cell-intrinsic, EGFR/ERBB inhibitor-induced IFN pathway activation in HNSCC and suggest that individual tumor responses to oncogene-targeted agents are a sum of direct growth inhibitory effects and variably-induced participation of host immune cells.

## Background

Treatment of head and neck squamous cell carcinoma (HNSCC) requires combined modalities of surgery, radiation and cytotoxic chemotherapy as well as targeted anti-epidermal growth factor receptor (EGFR) agents and most recently, immunotherapies that inhibit the PD1-PD-L1 axis. EGFR is overexpressed in ~ 90% of primary HNSCC [1, 2] and is a member of the erb-b2 receptor tyrosine kinase (ERBB) family of receptor tyrosine kinases (RTKs) that includes ERBB2-4. Despite only modest activity as single agents [3, 4], monoclonal antibodies targeting EGFR such as cetuximab and pan-EGFR/ERBB inhibitors continue to attract significant clinical attention in HNSCC [2, 4–6]. Moreover, based on sensitivity to EGFR- and pan-ERBB-specific TKIs, our previous studies identified subsets of EGFR/ERBB-dependent HNSCC cell lines and used functional genomics strategies to define EGFR, ERBB2 and ERBB3 as components of a non-mutated driver pathway [7–9]. Distinct from lung adenocarcinoma, the HNSCC genomic landscape has not unveiled gain-of-function mutations or gene amplification events that serve as biomarkers of clinical activity of cetuximab, either alone or when combined with chemotherapy or radiotherapy [3, 10–12]. The mechanisms accounting for the broad range of clinical response to EGFR-targeted inhibitors in HNSCC patients remain largely unknown and this knowledge gap represents a significant hurdle to their deployment as precision oncology agents.

Anti-PD1 antibodies, pembrolizumab [13] and nivolumab [14], have shown clinical efficacy in HNSCC and gained FDA approval [15]. In addition to agents that directly target host immunity, it is becoming clear that both cytotoxic and oncogene-targeted agents can induce mobilization of immune cells that are postulated to contribute both positively and negatively to the therapeutic response in HNSCC and other solid tumors [16–18]. The literature also demonstrates the ability of EGFR inhibitors to increase MHC expression and antigen presentation on tumor cells [19–21]. In fact, the ability of EGFR and MAPK pathway inhibitors to induce genes involved in antigen presentation likely reflects the activation of a larger interferon transcriptional program that, in addition to antigen presentation, includes a host of chemokines, cytokines and antiviral genes [22–27]. This literature is consistent with the ability of EGFR inhibitors to stimulate expression of various interferon (IFN)-stimulated genes in keratinocytes [24] and the observation that ablation of EGFR in mouse skin induces inflammatory responses [28, 29]. Importantly, overall survival with cetuximab treatment is greater in HNSCC patients experiencing an acneiform rash [30]. Combined, this literature supports the idea that EGFR/ERBB inhibition promotes the participation of the tumor immune microenvironment via induction of an interferon response program.

Individual HNSCC patients exhibit wide-ranging degrees of response to both cetuximab and ERBB family-targeted tyrosine kinase inhibitors (TKIs) such as afatinib [4], with only a fraction exhibiting significant tumor shrinkage. At present, the mechanisms underlying the heterogeneity of anti-EGFR therapy-induced tumor responses observed in HNSCC patients remain ill-defined. Certainly, the lack of biomarkers for predicting response to EGFR-targeted agents may contribute. Still, even within oncogene-defined subsets of lung cancers driven by mutant EGFR or ALK, the individual responses to targeted TKIs ranges widely. Moreover, a clear association exists between the initial degree of tumor shrinkage and progression free and overall survival in ALK + lung cancer patients treated with TKIs [31] and colorectal cancer patients treated with cetuximab [32, 33]. Thus, a deeper understanding of the biology underlying variation in response may unveil novel means to deepen tumor shrinkage responses with rational combinations of therapeutics. Herein, we measured transcriptional reprogramming in a panel of human HNSCC cell lines that exhibit equivalent growth sensitivity to EGFR/ERBB inhibitors and observed marked heterogeneity in both the magnitude and complexity of a TKI-induced interferon response program including diverse chemokines and cytokines that signal to immune cells in the tumor microenvironment [34–36]. We hypothesize that this TKI-induced, tumor cell-autonomous reprogramming response may variably influence the participation of the immune microenvironment in the therapeutic responses to EGFR antagonists within individual HNSCC tumors.

## Materials and methods

### HNSCC cell lines

*Human HNSCC cell lines.* Cal27, HN6, HN12, HN31, JHU-011, UMSCC8 and UMSCC25 cells were selected from our laboratory’s collection and cultured in Dulbecco’s modified Eagle’s medium (Invitrogen, Carlsbad, CA) supplemented with 5% fetal bovine serum and 1% penicillin–streptomycin (Sigma-Aldrich, St. Louis, MO) at 37 °C in a humidified 5% CO_2_ incubator. The cells used in this study were cultured from frozen stocks that had been submitted to fingerprint analysis to confirm authenticity.

*Murine HNSCC cells:* B4B8 squamous cell carcinoma cells were obtained from the lab of Dr. Nadarajah Vigneswaran (UTHealth, Houston, TX). B4B8 is a murine SCC cell line derived from BALB/c oral keratinocytes treated with chemical carcinogen 4NQO [37]. B4B8 is wildtype for TP53, KRAS, NRAS and EGFR. RNAseq analysis confirmed these genetic characteristics as a surrogate for verification of cellular authenticity. Cells were cultured in DMEM-F12 media (SigmaAldrich) supplemented with 5% FBS and 1% PS at 37 ºC and 5% CO_2_.

### In vivo mouse studies

Six-week-old female BALB/c mice were purchased from Charles River. For tumor cell inoculation, B4B8 cells were grown to 75% confluence and harvested and resuspended in serum-free DMEM media. Cell suspensions were mixed with equal volumes of Matrigel (10 mg/mL, BD Biosciences, San Jose, CA) and injected sub-mucosally via the intraoral route into the buccal mucosa at a final concentration of 2–3 × 10^6^/0.1 ml per animal. Forty mice were inoculated at a single site in the right buccal. Mice were randomized to receive AZD8931 (50 mg/kg; MedChem Express) by oral gavage daily for 3, 7, and 14 days. Treatment was started when average tumor size reached 30–50 mm^3^ (10–14 days post inoculation). Tumor size was measured with digital calipers and tumor volumes were estimated using the formula (V = A*B^2^/2 mm^3^), where A and B are the longer and shorter diameters of the tumor. Mice exhibiting signs of morbidity according to the guidelines set by the Institutional Animal Care and Use Committee (IACUC) were sacrificed immediately. Primary tumors, regional lymph nodes, spleens and lungs were harvested upon sacrifice. For histopathological and immunofluorescence (IF) studies, tissues were fixed in 10% neutral buffered formalin, embedded in paraffin and cut into serial sections. All protocols for animal tumor models were approved by the IACUC of the University of Colorado Denver.

### Cell culture

The human HNSCC cell lines Cal27, HN6, HN12, HN31, JHU011, UMSCC8 and UMSCC25 were cultured in Dulbecco’s Modified Eagle’s Medium (DMEM) (Invitrogen, Carlsbad, CA) supplemented with 5% fetal bovine serum (FBS) with 1% penicillin–streptomycin (Sigma-Aldrich, St. Louis, MO) at 37 °C in a humidified 5% CO_2_ incubator. All cancer cell lines used in this study were submitted to fingerprint analysis to confirm authenticity within a year of performing the studies described herein. The murine B4B8 cell line was a generous gift of Dr. Carter Van Waes (NIDCD, Bethesda, MD) and cultured in DMEM/F12 medium supplemented with 5% FBS and 1% PS.

### Affymetrix genechip analysis

UMSCC25 and HN31 HNSCC cells were treated with DMSO or 100 nM gefitinib for 4 days after which RNA was purified and used to probe Affymetrix Human Gene 1.0 ST arrays. Gene expression values were extracted and normalized by using Robust Multiarray Average (RMA) and Affymetrix Power Tools. Data analysis was performed on these normalized gene expression profiles.

### RNAseq and bioinformatics analysis

RNA was submitted to the University of Colorado Bioinformatics core where library prep was generated and RNA was sequenced on the NovaSeq 4000 to generate 2 × 151 reads. Fastq files were quality checked with FastQC, illumina adapters trimmed with bbduk, and mapped to the mouse mm10 genome with STAR aligner. Counts were generated by STAR’s internal counter and reads were normalized to counts per million (CPM) using the edgeR R package [38]. Differential expression was calculated using the limma R package and the voom() function [39]. Gene set enrichment analysis (GSEA) was performed using the fgsea R package (v1.10.0) [40] with hallmark gene sets from the Molecular Signatures Database [41] and 10,000 permutations. Heatmaps were generated in Prism 9 (GraphPad Software, San Diego, CA).

### Cell proliferation assay

Cells were plated at 100 cells per well in 96 well tissue culture plates and treated with inhibitors at various doses. When the DMSO-treated control wells became confluent (1–2 weeks), cell numbers were assessed using a CyQUANT Direct Cell Proliferation Assay (Invitrogen) according to the manufacturer’s instructions.

### Quantitative real-time PCR (qRT-PCR)

150000–400,000 cells were seeded in 10 cm plates and allowed to attach. After, 24 h, cells were treated with DMSO, 100 nM AZD8931, 500 nM ruxolitinib, 500 nM IKK16, or the combinations. Cells were collected in 600 µL RNA Lysis Buffer (RLB). Total RNA was purified from cells using Quick-RNA MiniPrep kits (Zymo Research, Irvine, CA) and aliquots (5 µg) were reverse transcribed in a volume of 20 µL using Maxima First Strand cDNA Synthesis Kit (Thermo Scientific, Pittsburgh, PA). Aliquots (2 µL) of a 1:5 dilution of the reverse transcription reactions were submitted to quantitative RT-PCR in 10 µL reactions with SYBR Select Master Mix for CFX (Thermo Fisher Scientific) using a CFX Connect Real-Time PCR Detection System (BioRad, Hercules, CA). The real-time PCR amplification products from initial experiments were resolved by electrophoresis on 5% polyacrylamide gels to verify that the primer pairs amplified a single product based on predicted sizes generated in MacVector. GAPDH mRNA levels were measured as a housekeeper gene for normalization of the different mRNA expression values, and the data are presented as “Relative Expression”.

### ELISA and luminex assays

#### ELISA

Conditioned media was collected from treated and untreated human and murine HSNCC cells. Chemokine levels were measured using the Invitrogen ELISA Kit (Quantikine mouse/human CXCL10/IP-10, IL6, TGFB2 ELISA kits; R&D Systems, Minneapolis, MN) following manufacturer’s instructions. Absorbance was measured at 450 nm. The measured concentration in each sample was normalized to the total cellular protein per dish and the data are presented as pg/µg.

#### Luminex assay

Cells were seeded in 10 cm dishes and 24-h later, the cells were treated with DMSO vehicle control or 100 nM AZD8931. The media from human and murine cell lines was collected 3 days later and assayed for human CXCL2, CXCL6, CXCL10, CCL5, CCL20, CSF1, CSF2, CSF3, IL1α, IL4, and OPN or murine CXCL1, CXCL2, CCL2, CCL5, CCL20, CCL22, CSF1, CSF2,and CSF3 according to the manufacturer’s instructions (Luminex kit; R&D Systems, Minneapolis, MN). The concentration of analyte in the media was normalized to the total cellular protein per dish and the data are presented as pg/µg.

### Dominant-negative IκB

The mutant IκB retroviral plasmid pQCXIP-mIκBα and pQCXIP empty vector control was kindly provided by Dr. Rebecca Schweppe (University of Colorado Anschutz Medical Campus) [42]. Both constructs were packaged in 293 T cells with retroviral packaging component vectors pSV-Ψ–env-MLV and pSV-Ψ–A-MLV. The retroviruses released into the medium were filtered using a 0.45 µm filter and used to transduce B4B8 cells in 10 cm plates at 500,000 cells/dish. Transduced cells were selected with puromycin (4 µg/ml) for 8 days to generate stable cell lines expressing dnIκB or pQCXIP.

### Immunofluorescence

Harvested tumor tissue was formalin-fixed and processed for paraffin embedding. For IF, 7 µm thick sections were deparaffinized with xylene and rehydrated with increasing concentrations of ethanol. Slides were incubated in 0.1% Sudan Black in 70% ethanol for 20 min at room temperature and heat-mediated antigen retrieval was performed using citrate buffer at low pressure for 2 h. Once cooled, slides were incubated in fresh 0.1 M glycine for 10 min followed by incubation with 10 mg/mL sodium borohydride on ice for 40 min. Tissues were blocked with goat-serum for 1 h and stained with CD3e (1:100; ThermoScientific #MA5-14524 clone SP7; rabbit anti-mouse) and CD4 (1:50; eBioscience #14-9766-82 clone 4SM95; rat anti-mouse) or CD8a (1:100; eBioscience #14-0808-82 clone 4SM15 rat anti-mouse) antibodies diluted in a 1:1 mixture of superblock and 5% BSA/TBST overnight at 4 °C. Secondary. Serial washes in TBST were used and slides were stained with secondary antibodies were used (CD3: AF594 goat anti-rabbit IgG A11012; CD4/CD8: AF488 goat anti-rat IgG A11006) at 1:5000 and incubated for 1 h at room temperature. ImageJ software version 1.8.0 was used to visualize images and perform data analysis.

### Multiplexed immunofluorescence staining of primary HNSCC patient specimens

NCT01218048 (UPCI 08-013) is a completed trial entitled “*Erbitux Followed by Adjuvant Treatment With Chemoradiation and Erbitux for Locally Advanced Head and Neck Squamous Cell Carcinoma*”. This phase II clinical trial of preoperative, single-agent cetuximab treated patients banked HNSCC tumor specimens before and after 4 weeks of cetuximab. Stage III/IV HNC patients (n = 22) were subsequently treated with definitive surgical resection and observed for disease recurrence. Cetuximab was administered for a 3–4-week preoperative period and the clinical response was recorded. We obtained sections of archival formalin-fixed, paraffin-embedded (FFPE) tissue on glass slide for immunofluorescence staining. Clinical response was previously analyzed by comparing paired CT scans pre- and post-cetuximab. “Responders” demonstrated reduction in tumor volume while “Non-responders” had tumors that grew during the course of therapy. Sections of primary HNSCC were stained using Opal multiplex according to manufacturer’s protocol (PerkinElmer) for DAPI, HLA-DR, CK, CD3, CD14, CD19 and CD8 by the Human Immune Monitoring Shared Resource at the CU Anschutz Medical Campus. Slide scanning was performed on the Vectra 3.0 instrument and five to fifteen multispectral regions of interest were selected per specimen with Phenochart 1.0.12. Images were spectrally unmixed and analyzed using inForm Software 2.4. Based on nuclear and membrane markers and phenotypically scored for CD3 + , CD4 (CD3 + , CD8−), CD8 (CD3 + CD8 +), CD14 + , CD19 + , CK + , and other (DAPI +).

### Quantification and statistical analysis

Statistics Analysis Prism 9 (GraphPad Software, San Diego, CA) was used to perform statistical analyses. Data are presented as the mean and S.D as indicated. An unpaired Student’s t test (two-tail) was used to determine statistical significance, unless otherwise noted. The P values are denoted by *(P < 0.05), **(P < 0.01), ***(P < 0.001), and ****(P < 0.0001) and were corrected for multiple comparisons (Dunnett).

## Results

### EGFR/ERBB inhibitors induce IFN response programs in a heterogeneous fashion in human HNSCC cell lines.

**Fig. 1:**
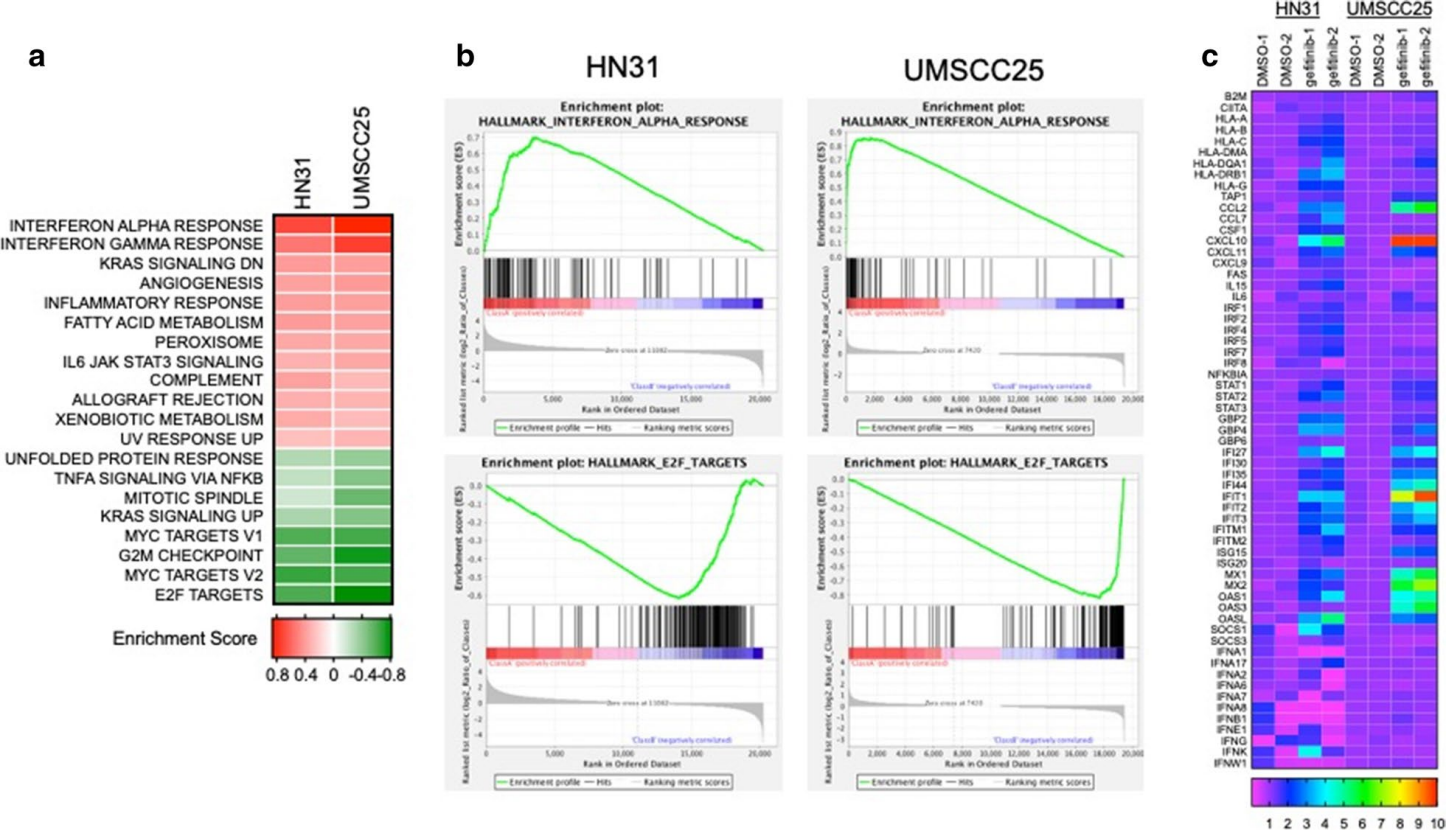
Gefitinib treatment transcriptionally induces interferon response hallmark pathways in human HNSCC cell lines. Total RNA was prepared from HN31 and UMSCC25 HNSCC cell lines treated in duplicate with DMSO or gefitinib (100 nM) for 4 days and submitted to Affymetrix Genechip analysis. **a** The resulting data were submitted to GSEA analysis using the Hallmark pathways and the enrichment scores for the two cell lines are presented as a heatmap. **b** The enrichment plots for the top enriched Hallmark pathway, the IFNα response, and the Hallmark pathway that exhibited the most negative enrichment in the gefitinib-treated samples, the E2F targets, are shown. **c** Selected individual genes from duplicate samples within the IFNα Hallmark pathway are presented as heatmaps with a rainbow scale depicting increased expression in response to gefitinib treatment

We recently defined EGFR-dependent human HNSCC cell lines based on sensitivity to EGFR-specific TKIs where the mean IC_50_ values for gefitinib and AZD8931 across the panel are 20.4 ± 3.1 and 4.1 ± 1.9, respectively [7–9, 43]. Two EGFR-dependent cell lines, UMSCC25 and HN31, were treated for 4 days with 100 nM gefitinib and RNA was submitted to Affymetrix gene expression profiling. Gene set enrichment analysis (GSEA) revealed enrichment of the IFNα and IFNγ Hallmark pathways and negative enrichment of proliferation-associated pathways including E2F targets, G2M checkpoint and MYC targets (Fig. 1a, b). Inhibited expression of these proliferation-associated gene sets is consistent with the strong growth inhibition observed in these cell lines in response to EGFR-specific TKIs [9]. By contrast, many of the genes within the IFN response pathways are innate immune genes typically involved in the antiviral response, but also include a host of chemokines and cytokines involved in communication with diverse immune cell types [44, 45] (Fig. 1c). Notably, none of the members of the interferon family of factors was induced at the mRNA levels in these cell lines, indicating that the mechanism by which gefitinib induced the IFNα and IFNγ pathways did not involve activation of IFN receptors through autocrine IFNs.

**Fig. 2:**
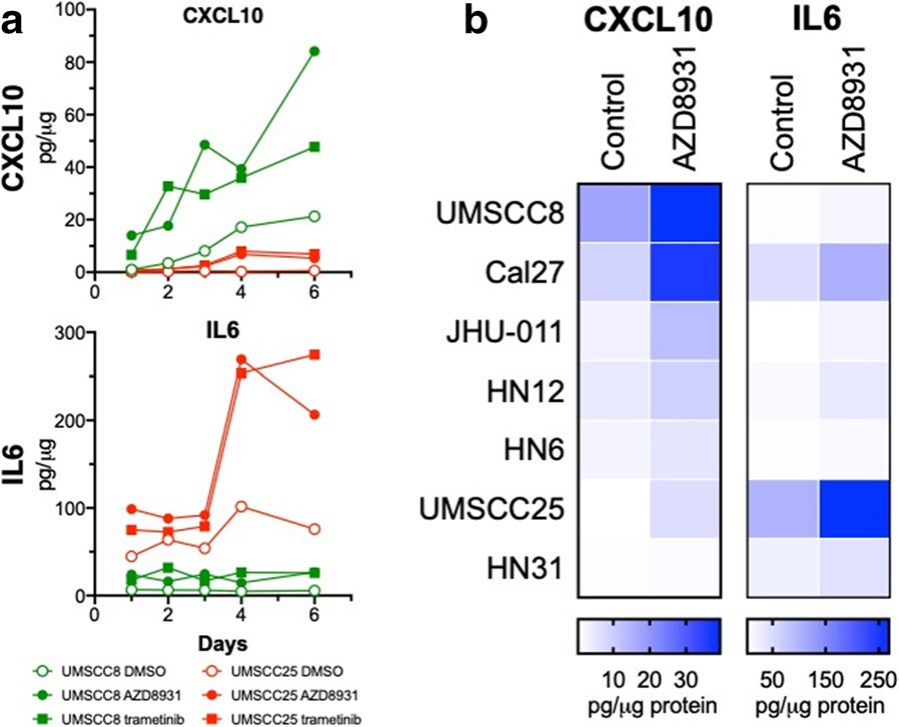
Variation in CXCL10 and IL6 induction by the pan-ERBB inhibitor, AZD8931, and MEK inhibitor, trametinib, in human HNSCC cell lines. **a** UMSCC8 and UMSCC25 cells were treated for 1 to 6 days with DMSO, AZD8931 (100 nM) or trametinib (10 nM) and conditioned media was analyzed by ELISA for CXCL10 and IL6. Levels were normalized to total cellular protein and presented as pg/µg protein. The data points represent single determinations at five distinct time points per treatment. **b** The indicated HNSCC cell lines were treated with DMSO or AZD8931 (100 nM) for 4 days and conditioned media was submitted to ELISA for CXCL10 and IL6. The color bar indicates the expression levels of CXCL10 and IL6 after normalization to cellular protein

To verify induction of these IFN response programs in response to EGFR inhibition, EGFR dependent HNSCC cell lines, UMSCC25 and UMSCC8, were treated with gefitinib over a time course of 2 h to 7 days. Total RNA was purified and submitted to quantitative RT-PCR assays for multiple genes within the IFN response programs. IFIT1, MX2, STAT1, and STAT2 mRNAs were all induced, with expression peaking at 3 days, in response to EGFR inhibition (Additional file 1: Fig. S1A). Moreover, the regulation of these genes by gefitinib was similar between the two cell lines. By contrast, several chemokines and cytokines were differentially regulated between UMSCC8 and UMSCC25. The levels of mRNAs for CXCL10, CCL28 and C3 were more highly induced by gefitinib in UMSCC8 cells while IL6, CCL5 and TGFβ2 mRNAs were more strongly regulated in UMSCC25 (Additional file 1: Fig. S1B). The difference in CXCL10, an anti-tumorigenic chemokine [46] and IL6, a pro-tumorigenic cytokine [47] is even more apparent when protein levels of these factors are measured by ELISA (Fig. 2a). CXCL10 protein was strongly induced by the pan-ERBB inhibitor, AZD8931, and the MEK inhibitor, trametinib, in UMSCC8 cells relative to UMSCC25 cells. By contrast, IL6 was markedly induced by the same agents in UMSCC25 cells compared to weak stimulation in UMSCC8 cells (Fig. 2a). Analysis of CXCL10 and IL6 secretion in a panel of EGFR-dependent human HNSCC cell lines that includes these two cell lines as well as five others (Cal27, HN6, HN12, HN31 and JHU-011) provided further evidence for marked heterogeneity of induction of these two IFN-stimulated genes among the seven cell lines (Fig. 2b). In addition to UMSCC8, Cal27 cells exhibited marked induction of CXCL10 protein in response to AZD8931 while HN6, HN12, JHU-011 and UMSCC25 showed more modest induction, and HN31 cells exhibited little or no CXCL10 induction. By contrast, IL6 was induced by AZD8931 in UMSCC25 and Cal27 cells. A more comprehensive Luminex analysis of chemokines and cytokines from Cal27, UMSCC8 and UMSCC25 cells (Additional file 1: Fig. S2A) validates the findings with CXCL10 in Fig. 2. Also, the data indicate the heterogeneity of the TKI-induced chemokine profile in distinct cell lines. For example, CCL5 and CSF1 are induced by AZD8931 in all three cell lines while CXCL2 is induced only in Cal27 and UMSCC25 and CXCL6 is induced only in Cal27 and UMSCC8 cells. Combined, the findings show that complex and variable mixtures of chemokines and cytokines with distinct roles as immune regulatory factors are produced in response to AZD8931 treatment in a cell line-dependent manner.

### Murine HNSCC cells exhibit EGFR/ERBB-dependency and increased transcription of IFN response programs upon EGFR/ERBB inhibition.

**Fig. 3:**
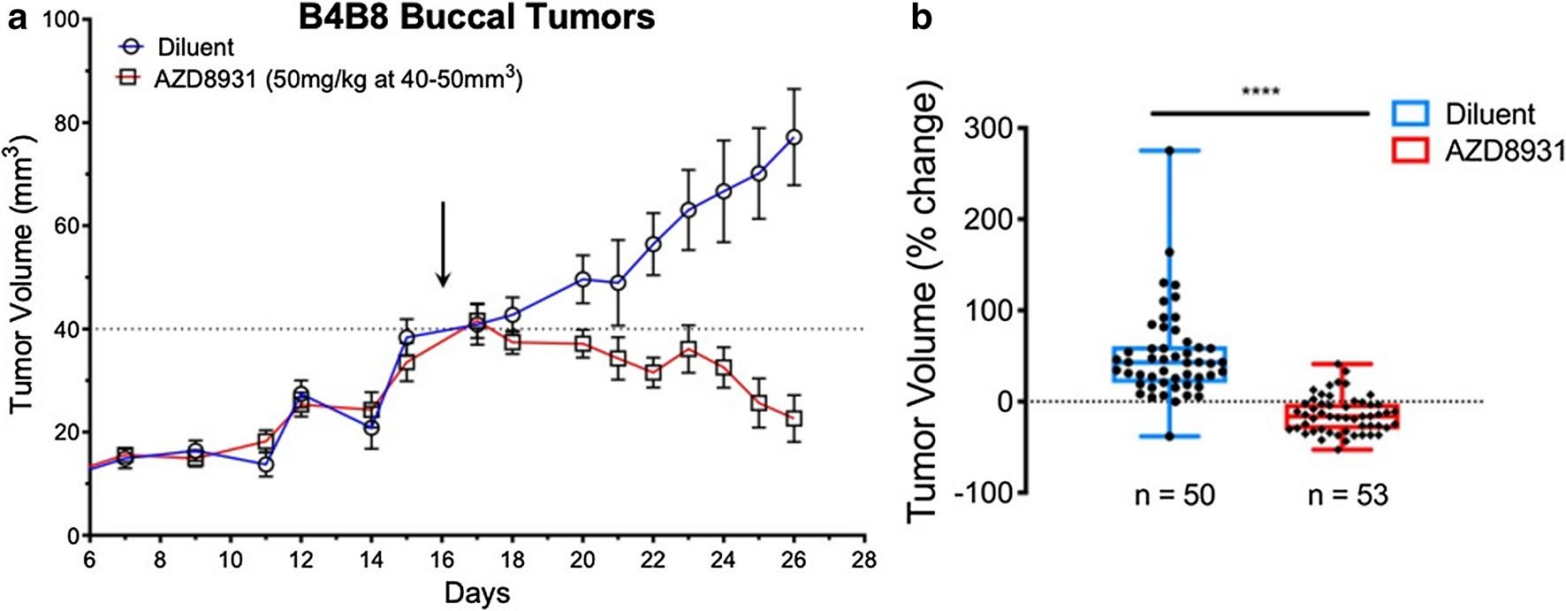
Response of orthotopic B4B8 murine HNSCC tumors to AZD8931. B4B8 cells were inoculated into the buccal space of BALB/c mice as described in the Materials and Methods. When tumors reached 40 to 50 mm^3^ (~ 15 to 16 days after inoculation), mice were randomized for daily gavage with diluent or 50 m/kg AZD8931. Tumor diameter was measured with calipers and volume was calculated as described. **a** Time course of growth of buccal B4B8 tumors treated daily by oral gavage with diluent control (n = 50) or 50 mg/kg AZD8931 (n = 53). The data are the mean and SEM of three independent compiled experiments. **b** The therapeutic responses of individual B4B8 tumors after 7 days of diluent or AZD8931 treatment are presented as the percent change from initial tumor volume

In addition to human HNSCC cell lines, we surveyed available murine HNSCC cells lines that can be propagated in syngeneic hosts for sensitivity to EGFR/ERBB inhibitors. Among the 4 murine HNSCC cell lines tested, B4B8 [37] is dependent on EGFR/ERBB signaling for growth as assessed by sensitivity to the pan-ERBB inhibitor, AZD8931, and the specific EGFR inhibitor, gefitinib (Additional file 1: Fig. S3). In fact, the sensitivity of B4B8 cells to gefitinib and AZD8931 is similar to that observed in human EGFR-dependent HNSCC cell lines [9]. To define the sensitivity of B4B8 tumors propagated in syngeneic hosts, B4B8 cells (2–3 × 10^6^) were injected orthotopically into the buccal space of immune competent BALB/c mice. When tumors reached 40–50 mm^3^ (~ 16 days), mice were treated daily with AZD8931 (50 mg/kg) or diluent by oral gavage. As shown in Fig. 3a, treatment with AZD8931 rapidly halts B4B8 tumor progression and causes significant shrinkage of tumors (Fig. 3b) compared to tumor-bearing mice treated with diluent as a control. Thus, B4B8 cells serve as a murine HNSCC model to explore EGFR/ERBB-dependent growth in an in vivo setting.

**Fig. 4:**
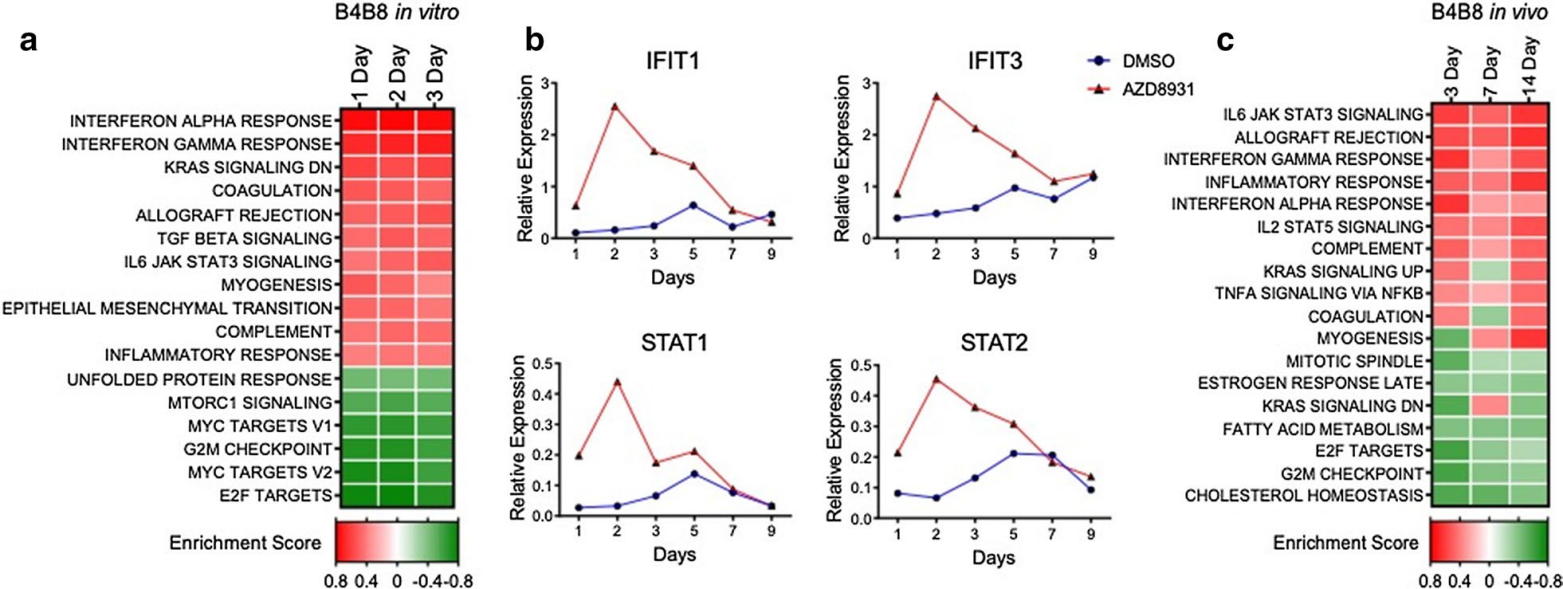
IFN pathway activation by EGFR/ERBB inhibitor in murine B4B8 HNSCC cells. **a** RNA was purified from B4B8 cells treated in vitro with DMSO or AZD8931 (100 nM) for 1, 2 or 3 days and submitted for RNAseq. The resulting data were analyzed with GSEA and the Hallmark Pathways. **b** B4B8 cells were treated for 1–9 days with DMSO or AZD8931 (100 nM) and RNA was purified and submitted to RT-QPCR for the indicated IFN pathway genes. **c** GSEA analysis of RNAseq from B4B8 tumors treated in vivo with diluent or AZD8931 (50 mg/kg) for 3 (n = 2), 7 (n = 5) and 14 (n = 4) days. The heatmap presents the enrichment or de-enrichment of the Hallmark pathways that exhibited the highest or lowest significant (P ≤ 0.05) enrichment scores

**Fig. 5:**
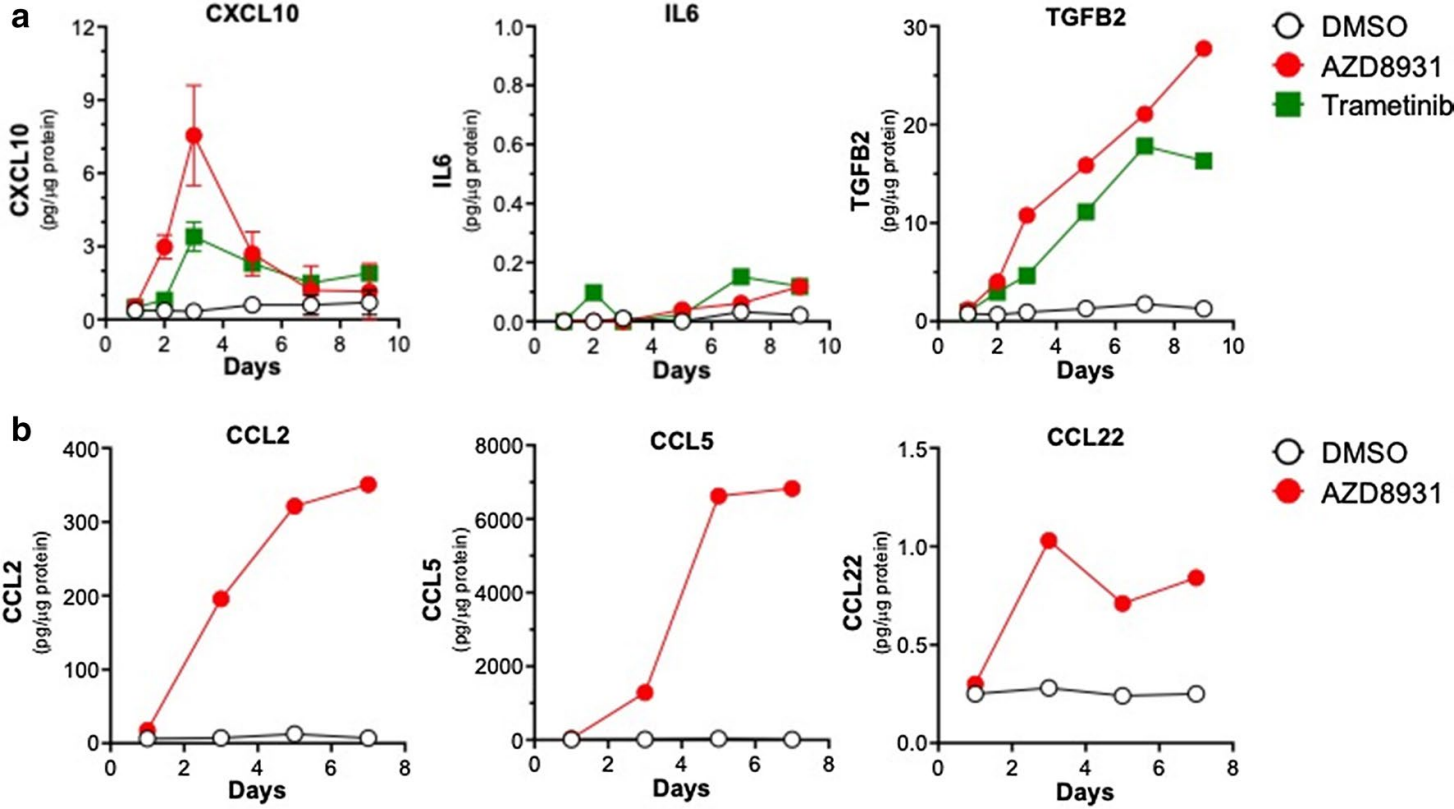
Induction of chemokine and cytokine expression by EGFR/ERBB and MEK inhibitor treatment in B4B8 cells.** a** B4B8 cells were treated with DMSO, AZD8931 (100 nM) or trametinib (10 nM) for 1–9 days. Conditioned media was collected and submitted to ELISA for CXCL10, IL6 and TGFB2. The data are the means and SEM three independent experiments (CXCL10) or single determinations at 6 distinct time points (IL6 and TGFB2) and presented as pg per µg of cellular protein. **b** B4B8 cells were treated for the indicated time with DMSO or AZD8931 (100 nM) and conditioned media was submitted to Luminex analysis for the indicated secreted analytes. The data points represent single determinations at four distinct time points per treatment

To explore transcriptional reprogramming induced by AZD8931 in B4B8 cells, total RNA was isolated from B4B8 cells treated in vitro for 1–3 days with AZD8931 or DMSO and submitted to RNAseq. GSEA showed significant enrichment of the same hallmark IFNα and IFNγ response programs (Fig. 4a) as observed in the human HNSCC lines (Fig. 1a). AZD8931-induced expression of specific genes that comprise the IFNα and IFNγ response programs including genes with functions in innate immunity (IFIT1, IFIT3, STAT1, STAT2; Fig. 4b) as well as multiple secreted chemokines (Fig. 5, Additional file 1: Fig. S2B) were observed. Similar to the human HNSCC cell lines, both AZD8931 and trametinib stimulated expression of CXCL10 in B4B8 cells (Fig. 5a), indicating that inhibition of the MAPK signaling pathway is central to the observed transcriptional responses. Notably, MOC2 cells that are insensitive to EGFR/ERBB inhibitors (Additional file 1: Fig. S3) exhibit increased expression of IFIT3, MX2, STAT1 and STAT2 with trametinib treatment, but not AZD8931 (Additional file 1: Fig. S4), demonstrating that the actions of AZD8931 are linked to the dependency of B4B8 cells on ERBB family RTKs. To determine if B4B8 tumors treated in vivo with AZD8931 show changes in IFN pathway activity, tumor-bearing mice were sacrificed after 3, 7, and 14 days of treatment, RNA was isolated and submitted to RNAseq. GSEA showed enrichment of the IFNα and IFNγ response programs (Fig. 4c), as also observed in vitro with human and mouse HNSCC cell lines (Figs. 1, 4a).

### IKK-NFκB and JAK signaling is required for EGFR inhibitor-induced IFN pathway responses

Diverse signal pathways function proximally to the induction of IFN programs and include JAK/STAT-coupled IFN receptors, NFκB-linked receptors as well as the STING and double-stranded RNA-coupled mechanisms [48–50]. To define pathways responsible for innate immune gene and chemokine induction, the human HNSCC cell lines UMSCC8 and UMSCC25 were treated with gefitinib in combination with either a JAK/STAT inhibitor, ruxolitinib, or an IKK/NFκB inhibitor, IKK16. These treatment combinations reduced AZD8931-induced mRNA expression of innate immune genes and chemokines, although in a distinct manner between the two cell lines (Additional file 1: Fig. S5A). For example, IFIT1, MX2, STAT1 and STAT2 mRNA induction was largely sensitive only to ruxolitinib in UMSCC8 cells, but sensitive to both ruxolitinib and IKK16 in UMSCC25 cells (Additional file 1: Fig. S5A). In UMSCC8 cells, induction of CXCL10 mRNA and protein by AZD8931 and MEK inhibitor trametinib are largely IKK16 sensitive (Additional file 1: Fig. S5A and B). Furthermore, in UMSCC8 cells, CCL5 mRNA induction is sensitive to both ruxolitinib and IKK16 and C3 only sensitive to ruxolitinib. In UMSCC25 cells, the more modest induction of CXCL10 mRNA by gefitinib (Additional file 1: Fig. S5A) and protein by AZD8931 (Additional file 1: Fig. S5B) is reduced by ruxolitinib and IKK16 while IL6, TGFB2 and CCL5 mRNA induction are reduced only by IKK16. Similar to UMSCC25 cells, AZD8931 induced CXCL10 protein in murine B4B8 HNSCC cells was blocked by both ruxolitinib and IKK16 (Additional file 1: Fig. S5C). Consistent with a general dependence on IKK, increased NFκB activity was demonstrated in B4B8 cells following transfection of a NFκB-responsive firefly luciferase reporter (Additional file 1: Fig. S5D) where IKK16, but not ruxolitinib, reduced AZD8931-induced reporter activity. Finally, B4B8 cells stably transduced with a dominant-negative IκB construct that blocks NFκB translocation to the nucleus, strongly inhibited AZD8931, gefitinib and trametinib-induced expression of CXCL10 protein (Additional file 1: Fig. S5E). Overall, these findings reveal dependence on NFκB and JAK pathways for EGFR/ERBB and MEK inhibitor-induced IFN pathway gene expression, although the precise requirements are gene and cell line specific.

### AZD8931 stimulates CD8 + T cell accumulation in B4B8 tumors and therapeutic response is diminished in immune-deficient mice

**Fig. 6:**
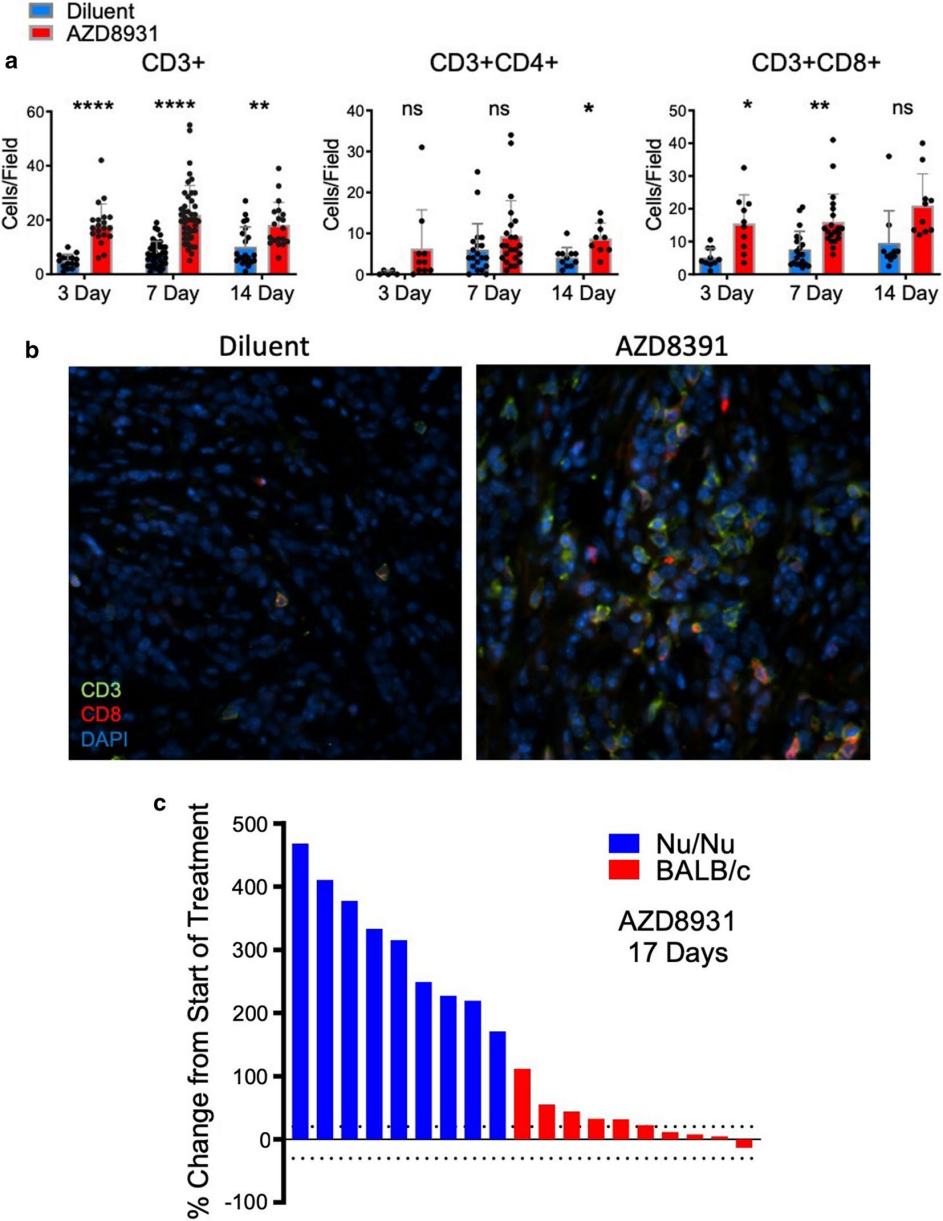
AZD8931 increases CD8 + T cell content in B4B8 tumors and therapeutic activity is diminished in immune-deficient mice.** a** B4B8 tumors treated with diluent or AZD8931 (50 mg/kg) for 3 (control, n = 1–2; treated, n = 2), 7 (control, n = 4; treated, n = 4), or 14 (control, n = 2; treated, n = 2) days were formalin fixed, paraffin embedded and submitted to immunofluorescence staining for the T cell marker CD3e with CD4 or CD8α. Five ROIs per tumor were randomly selected (accounting for > 70% of the section area). Two independent observers quantified each field of view for the number of positively stained cells per field and the average of the two independent observations and SD are reported. **b** Representative immunofluorescence images of CD3e + (green), CD8α + (red) and DAPI stained B4B8 tumors treated with diluent or AZD8931 (50 mg/kg). **c** B4B8 tumors were implanted in nu/nu mice or syngeneic BALB/c mice and treated with AZD8931 (50 mg/kg). The percent change in tumor volume of individual tumors after 17 days of treatment with AZD8931 in BALB/c and nu/nu mice is presented as a waterfall plot

A predicted result of EGFR/ERBB inhibitor-induced expression of chemokines and cytokines in human and murine HNSCC cell lines would be treatment-induced immune cell infiltration. To explore changes in immune cell content within murine HNSCC tumors, B4B8 tumors treated for 3, 7 and 14 days with AZD8931 were stained for CD3e and either CD4 or CD8α by immunofluorescence. CD3 + cells were significantly increased at all times following AZD8931 treatment (Fig. 6a). CD3 + CD8 + T cells were significantly induced at the 3 and 7-day timepoints while CD3 + CD4 + T cells were not significantly increased until 14 days of TKI treatment (Fig. 6a, b). The findings support that AZD8931 treatment of B4B8 tumors stimulates infiltration of T cell populations early in the course of treatment.

To determine if the increase in T cells contributes to the therapeutic response to AZD8931, B4B8 cells were orthotopically implanted into the buccal space of nu/nu mice which lack functional T and B cells as well as immune competent BALB/c mice. Tumors were permitted to establish for 12 days and then all mice were treated daily with AZD8931 (50 mg/kg) by oral gavage. The individual responses of the tumors (percent of initial) to 17 days of AZD8931 treatment in BALB/c and nu/nu mice are presented in Fig. 6c as a waterfall plot and demonstrate the marked difference in AZD8931 responsiveness between the two murine host systems. These data support the hypothesis that EGFR/ERBB inhibitor-induced mobilization of the host immune system contributes to therapeutic efficacy in this murine model of HNSCC.

### Clinical response to cetuximab in HNSCC patients is positively associated with increased CD8 + T cell content.

**Fig. 7:**
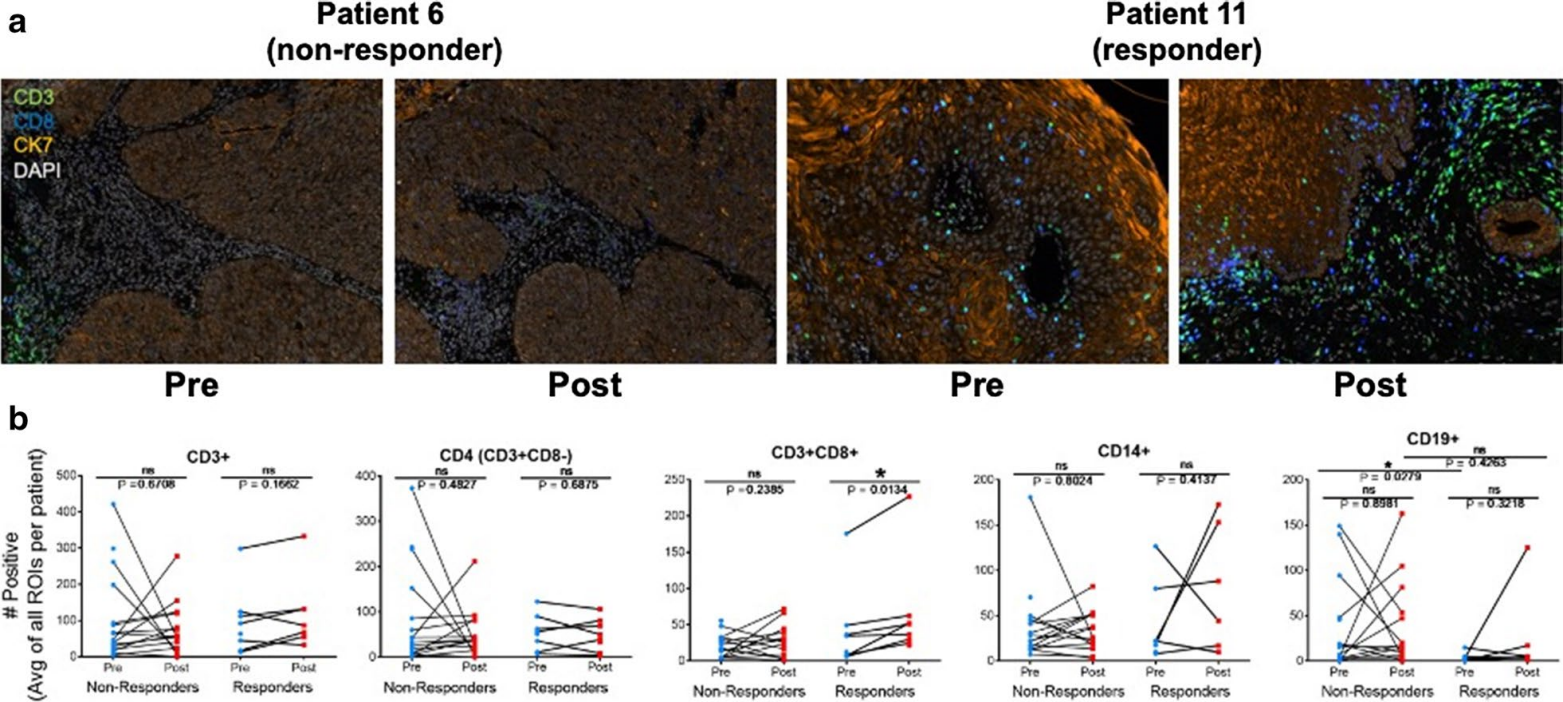
Immune cell alterations associated with cetuximab treatment in HNSCC patients. **a** 22 matched pairs (15 pairs were from non-responders and 7 pairs were from responders) of pre-treatment biopsies and on-treatment (cetuximab) specimens were obtained as FFPE sections on glass slides. Representative images of Vectra 3.0 staining of patient tumors before and following 3–4 weeks of cetuximab treatment. **b** InForm analysis of immune cell content in primary HNSCC pre- and post-cetuximab treatment. Each data point is the mean of 5–15 distinct regions of interest within each tumor section and the resulting data were submitted to a paired T test

To explore EGFR/ERBB inhibitor-induced immune cell mobilization and therapeutic response in HNSCC patients, specimens were obtained from a completed neoadjuvant cetuximab trial (UPCI-08–013) entitled “*Erbitux followed by Adjuvant treatment with chemoradiation and erbitux for locally advanced head and neck squamous cell carcinoma*” (NCT01218048) [51]. Sections of 22 matched biopsy pairs obtained from HNSCC patients prior to and after 3–4 weeks of neoadjuvant cetuximab therapy were stained using the Vectra 3.0 platform for the tumor marker cytokeratin 7 (CK7), the T cell markers, CD3 and CD8, the monocyte/macrophage marker, CD14, and the B cell marker, CD19 (see Materials and Methods). CD4 + T cells were inferred from subtracting CD3 + CD8 + cells from total CD3 + cells. Of the 22 patients, 7 exhibited a good response to cetuximab and 15 exhibited a poor response. Figure 7a shows representative fields from a non-responder (patient 6) and responder (patient 11) and stained for CK7, CD3 and CD8. In Fig. 7b, the means of the 5–15 independent fields analyzed per pre- and post-treatment specimen are graphed. No statistically significant changes in numbers of CD3 + and inferred CD4 + T cells were observed in pre- and post-cetuximab treatment specimens regardless of the treatment response. However, numbers of CD3 + , CD8 + T cells were significantly increased in post-treatment specimens within the responder group, but not the non-responder group (Fig. 7b). While numbers of CD14 + and CD19 + cells did not change between pre- and post-cetuximab treated samples regardless of responder status, the pre-treated biopsies of the non-responder patients exhibited significantly greater number of CD19 + B cells compared to responders (Fig. 7b). The analysis indicates that increased content of CD8 + T cells following 3–4 weeks of cetuximab treatment is associated with a therapeutic response to EGFR antagonists, like cetuximab.

## Discussion

A major focus of this study was to investigate the tumor cell autonomous effects of inhibiting the EGFR/ERBB signaling cascade in HNSCC. Herein, we showed that human and murine EGFR/ERBB-dependent HNSCC cell lines treated with specific TKIs undergo transcriptional induction of an IFN response program comprised of diverse genes with antiviral function and distinct chemokines and cytokines that communicate with the immune microenvironment. Despite similar growth sensitivities to EGFR/ERBB inhibitors [9], the quantitative and qualitative nature of the response varied markedly amongst the different human HNSCC cell lines. With regard to chemokine and cytokine expression, cell line-specific variation in both the magnitude and kinetics of induction by EGFR/ERBB inhibitors was observed. For example, UMSCC8 cells exhibited strong induction of the T cell chemoattractant, CXCL10, while UMSCC25 cells exhibited marked induction of the immunosuppressive chemokine, IL6 (Fig. 2, Additional file 1: Fig. S1). Based on the unique functions of these two factors as well as other chemokines measured in this study [36], distinct and widely-varying integrated activity on the immune microenvironment and overall therapeutic response to the EGFR/ERBB inhibitor might be predicted in vivo. We postulate that the HNSCC cell lines provide models for understanding the heterogeneous response to cetuximab and afatinib therapy observed in cohorts of HNSCC patients [4]. Specifically, tumors that exhibit significant shrinkage may more strongly induce IFN pathway responses due to therapy-mediated secretion of chemokines that recruit antitumor immune cells (modeled by UMSCC8 cells) compared to tumors that exhibited an immune suppressive response to therapy (UMSCC25) and progressed on treatment.

The literature supports the ability of oncogene-driven pathways to negatively influence immunological function such that oncogene-targeted agents unleash an IFN response arising within the tumor cells [16, 26, 27, 52]. While our study focused on chemokines and cytokines regulated in response to EGFR/ERBB inhibition in HNSCC cells, Pollack et al. showed that EGFR inhibition can augment expression of CIITA and antigen presenting machinery (APC) [19, 20]. In support, our human HNSCC cell lines also showed induction of MHC class I (B2M) and MHC class II (HLA-DM) genes in response to ERBB inhibition (Additional file 1: Fig. S6 A). Likewise, treatment of murine B4B8 HNSCC cells with AZD8931 induced classic MHC class I and II genes (Additional file 1: Fig. S6 B) as well as specific BALB/c MHC class I and II genes (Additional file 1: Fig. S6 C). Thus, EGFR/ERBB inhibition in HNSCC cells results in elaboration of chemokines and cytokines involved in paracrine signaling to the immune microenvironment as well as increased expression of proteins that function to present neoantigens to T cells. Some published studies indicate that induction of chemokines and cytokines in response to EGFR and MEK inhibitors requires co-stimulation with IFNγ and TNFα [53, 54]. Yet, our studies demonstrate that multiple chemokines and cytokines are induced to varying degrees among multiple cell lines with EGFR/ERBB inhibition alone. Certainly, the kinetics of induction of the response is dynamic such that analysis at a single time point may miss peak responses. Also, the degree of IFN response induction by EGFR/ERBB inhibitors across the panel of human HNSCC cell lines varies widely (Fig. 2, Additional file 1: Fig. S2). Indeed, Kang et al. observed a weaker trametinib induction of CXCL10 secretion in HN31 cells relative to the other HNSCC cell lines investigated [54]. Similarly, our studies demonstrate that CXCL10 induction in HN31 cells is the least among the 7 cell lines assayed in Fig. 2b. While we did not explore the induction of the IFN program by combinations of EGFR/ERBB inhibitors and IFNγ or TNFα, it is likely that additive or synergistic activation of this pathway occurs and may more fully capture the response to oncogene inhibitors observed in vivo where paracrine interactions between tumor cells and immune cells can occur.

Our findings reveal EGFR/ERBB inhibitor-stimulated influx of T cells into orthotopic B4B8 tumors propagated in syngeneic mice and reduced anti-tumor activity of AZD8931 on tumors propagated in immune-deficient mice (Fig. 6c). These results support a hypothesis that transcriptional induction of an IFN response within tumor cells signals the contribution of host immunity to the observed therapeutic response. In support, Kuske et al. reported several published studies that show oncogene-targeted therapies in melanoma and breast cancer can affect the immune tumor microenvironment, where MEK or BRAF inhibitors resulted in production of immune stimulatory cytokines (IFNγ, TNFα, IL12) capable of recruiting and activating anti-tumorigenic T cells, or by reducing immunosuppressive cytokines (IL6 and CCL2) that keep pro-tumorigenic cell types, such as MDSCs, at bay [55]. Furthermore, the therapeutic effect of these targeted inhibitors was shown to be associated with the presence or absence of these immune cell subsets. The immunological effects of targeted therapy have also been documented in HNSCCs where inhibition of the mTOR, VEGF, and CDK pathways have been shown to promote anti-tumor immunity, including the recruitment of T cells [16]. To study the immunological effects of targeted therapy in HNSCC patients, investigators at the University of Pittsburg executed a window of opportunity trial (UPCI-08-013) to explore the effect of single agent cetuximab treatment on immune biomarkers and if changes in immunological subsets are associated with a reduction in tumor volume. Comparing patients based on their response, cetuximab treated tumors showed significantly increased monocytic MDSC in non-responders, while responders displayed decreased granulocytic MDSC and monocyte populations with attenuated M2 polarization [51]. Additionally, follow up studies reported that cetuximab responders exhibited upregulation of CD137, a marker of NK cell activation, on tumor infiltrating NK cells and an increase in frequency of circulating EGFR-specific T cells between cetuximab-treated and cetuximab-naive patients [56]. In agreement with these findings, our study found a significant increase in the number of CD8 + T cells in on-treatment specimens from cetuximab responders, while those that were defined as non-responders did not. Overall, the data from these specimens provide important insight into the distinct and variable response of individual HNSCC tumors, within the setting of single agent cetuximab therapy, that can be attributed to the differences in establishing pro- or anti-tumorigenic immune microenvironments.

HNSCCs typically present immunosuppressive tumor microenvironments and recurrence has been attributed to the establishment of immune evasion mechanisms such as upregulated expression of immune check point receptors [16]. Monoclonal antibodies targeting the PD-1/PD-L1 signaling axis, pembrolizumab and nivolumab, are now FDA approved, yet similar to cetuximab, less than 20% of HNSCC patients exhibit clinical benefit [13, 14]. Due to cetuximab’s documented stimulatory effects on the immune system, there are currently over 20 studies planned or underway to investigate the safety and efficacy of cetuximab immunotherapy combinations, including anti-PD1/PDL1/CTLA-4 (T cell modulation), TLR agonists, anti-NKG2A (NK cell modulation) and recombinant IL12 [16]. Completed studies with TLR8 agonists did not show increased clinical efficacy [57] while others with TLR9 agonist, motolomid, have shown increased NK responsiveness to anti-NKG2a suggesting potential synergy with cetuximab treatment [58]. Ferris et al. (NCT01935921) investigated motolimod or placebo in combination with EXTREME (platinum, fluorouracil, cetuximab) and observed increases in PFS and OS only in HPV-positive patients and those that had injection site reactions [16]. Lastly, a phase 2 clinical trial currently underway is investigating the ORR of pembrolizumab (anti-PD1) combined with cetuximab (NCT03082534). While the majority of the studies have focused on cetuximab’s ability to modulate the immune microenvironment due to the effector function intrinsic to the monoclonal antibody, herein we provide evidence that inhibition of the EGFR/ERBB receptors can contribute to immune stimulatory effects via transcriptional induction of an IFN response. Importantly, the magnitude of the IFN response varies widely among HNSCC cell lines (Fig. 2 and Additional file 1: Fig. S1 and [53, 54]. We hypothesize that this tumor cell-intrinsic property may contribute to the heterogeneity of EGFR/ERBB inhibitor responses observed in HNSCC patients. Moreover, the repertoire of chemokines and cytokines induced in response to EGFR/ERBB inhibition varied significantly among the human HNSCC cell lines (see Fig. 2 and Additional file 1: Fig. 2). Because distinct chemokines and cytokines recruit immune cell populations with either pro- or anti-tumorigenic function [36] the varying repertoire of chemokines and cytokines produced in response to EGFR/ERBB inhibition is predicted to yield actions on the immune microenvironment ranging from strongly anti-tumorigenic to highly immunosuppressive. Thus, as an alternative to combinations of EGFR/ERBB inhibitors with existing anti-PD1/PD-L1 agents, combining EGFR/ERBB inhibitors with novel agents that either enhance production of T cell attractants (like CXCL10) or inhibit production of chemokines/cytokines that recruit pro-tumorigenic immune cells like MDSCs may represent a next generation of therapeutic strategies in HNSCC. In this regard, preclinical studies have shown the capacity of antibodies targeting chemokine receptors, intra-tumoral injection of recombinant proteins (CXCL9/10), or epigenetic modifiers that dis-inhibit chemokine expression to attract immune cells capable of reducing tumor burden and prolonging survival in various murine cancer models [46, 59, 60].

## Conclusion

In summary, the results demonstrate a wide range of EGFR/ERBB inhibitor-induced interferon program activation in human and murine HNSCC cell lines and support the hypothesis that this transcriptional response variably instructs participation of the immune microenvironment in the overall therapeutic response. The findings may extend generally to the wide range of clinical activity observed with precision oncology drugs and future studies may unveil approaches to broaden oncogene inhibitor-stimulated immune surveillance for deeper and more prolonged clinical benefit in patients.

## Supplementary Information

**Additional file 1: Figure S1. **Kinetics of gefitinib-stimulated interferon response gene induction in UMSCC8 and UMSCC25 cells. Total RNA was purified from UMSCC8 and UMSCC25 HNSCC cells treated for 2 hrs to 7 days with DMSO or gefitinib (300 nM) and submitted to RT-QPCR analyses for **A**, IFIT1, MX2, STAT1 and STAT2 with roles in innate immunity and **B**, the indicated chemokines and cytokines. The mRNA expression levels were normalized to GAPDH mRNA levels and presented as Relative Expression. The data points represent single determinations at six distinct time points per treatment. **Figure S2. **Multiplex analysis of chemokine and cytokine induction in HNSCC cell lines in response to the pan-ERBB inhibitor, AZD8931**. A**, The indicated human HNSCC cell lines were treated for 3 days with DMSO or 100 nM AZD8931 and conditioned media was analyzed in duplicate using a multiplexed Luminex assay for distinct secreted factors (see Materials and Methods). Expression levels for the analytes were normalized to the maximum measurement within the three cell lines and the data are presented as a heat map. Samples that were above or below the detection limit of the assay are indicated in grey. **B**, B4B8 cells were treated for 3 days with DMSO or AZD8931 (100 nM) and media was collected for a Luminex multiplexed assay for murine chemokines and cytokines. The data are presented as fold-stimulation by AZD8931 relative to DMSO treated cells. **Figure S3. **Sensitivity of murine HNSCC cell lines to EGFR/ERBB inhibitors. The murine HNSCC cell lines B4B8, MOC1, MOC2 and LY2 were submitted to clonogenic growth assays with triplicate determinations at each concentration of **A**, AZD8931 or **B**, gefitinib. **Figure S4**. Innate immune gene regulation by trametinib, but not AZD8931 in murine MOC2 HNSCC cells. MOC2 cells were treated for 3 days with DMSO, AZD8931 (100 nM) or trametinib (30 nM). RNA was purified and submitted to RT-QPCR for the innate immune genes IFIT3, MX2, STAT1 and STAT2 and expression was normalized to GAPDH mRNA levels. The data points represent single determinations at three distinct time points per treatment. **Figure S5. **EGFR/ERBB inhibitor-induced IFN pathway activation is dependent on IKK/NFκB and JAK signaling in human and murine HNSCC cell lines. **A**, UMSCC8 and UMSCC25 cells were treated for 3 days with DMSO or gefitinib (300 nM) alone or in combination with ruxolitinib (1 uM) or IKK16 (500 nM). RNA was purified and submitted to RT-QPCR for the indicated genes and normalized to GAPDH mRNA levels. The maximum expression level for each gene among the two cell lines was used to normalize the distinct genes to a value of 1 and the data were presented as a heat map. **B**, UMSCC8 and UMSCC25 cells were treated for 3 days with DMSO, AZD8931 (100 nM) or trametinib (10 nM) in the presence or absence of ruxolitinib or IKK16. Conditioned media was collected and submitted to ELISA for human CXCL10. The data are the mean and SD of three independent experiments and presented as pg CXCL10 per µg of cellular protein. **C**, B4B8 cells were treated for 3 days with DMSO, AZD8931 (100 nM) or gefitinib (300 nM) in the presence or absence of ruxolitinib (1 µM) or IKK16 (500 nM). Conditioned media was collected and submitted to ELISA for murine CXCL10. The data are the mean and SD of three independent experiments and presented as pg CXCL10 per µg of cellular protein. **D**, B4B8 cells were transfected with an NFκB-responsive firefly luciferase reporter plasmid and a thymidine kinase-driven renilla luciferase reporter to estimate transfection efficiency. Following a 24-hour incubation, the transfected cells were treated with DMSO or AZD8931 alone or in combination with IKK16 or ruxolitinib. The data are the mean and SD of 3 independent experiments, and presented as fold-stimulation relative to DMSO treated cells. **E**, B4B8 cells were transduced with a retroviral vector encoding a dominant-negative IκB construct or an empty vector as a control (see Materials and Methods) and selected for puromycin resistance. The resulting cultures were treated for 3 days with DMSO, AZD8931 (100 nM), gefitinib (300 nM) or trametinib (10 nM) and conditioned media was submitted to ELISA for murine CXCL10. The data are the mean and SD of three independent experiments. **Figure S6. **EGFR/ERBB inhibition augments expression of antigen presentation genes in human and murine HSNCC cell lines *in vitro*. **A, **The human HNSCC cell lines UMSCC8 and UMSCC25 were treated with the EGFR inhibitor gefitinib (300 nM) over a time course of 1 to 7 days. RNA was submitted to RT-QPCR analyses for the MHC class I (B2M) and II (HLA-DMA) genes. The mRNA expression levels were normalized to GAPDH mRNA levels and presented as Relative Expression.** B, **The murine HNSCC cell line B4B8 was treated with the pan-ERBB inhibitor AZD8931 (100 nM) over a time course of 1 to 3 days and RNA was submitted to sequencing and expression of multiple MHC class I and II genes are reported as CPM. **C, **B4B8 cells were treated *in vitro* with DMSO or AZD8931 (100 nM) for 3 days, stained with PE-labeled anti-mouse MHC Class I (H-2Kd, H-2Dd; Invitrogen Clone 34-1-2S) or APC-labeled anti-mouse MHC Class II (I-Ad; eBioscience Clone AMS-32.1) and submitted to flow cytometry analysis. The median intensity of the fluorophore is presented, and the data are the mean of 2 independent experiments.

## Data Availability

The datasets used and/or analyzed during the current study are available from the corresponding author on reasonable request.

## Abbreviations

HNSCC: Head and neck squamous cell carcinoma
EGFR: Epidermal growth factor receptor
GSEA: Gene set enrichment analysis
qRT-PCR: Quantitative real-time PCR
TKI: Tyrosine kinase inhibitor
RTK: Receptor tyrosine kinase
IFN: Interferon

## References

[1] Grandis JR, Tweardy DJ (1993). Elevated levels of transforming growth factor alpha and epidermal growth factor receptor messenger RNA are early markers of carcinogenesis in head and neck cancer. Cancer Res.

[2] Bossi P, Resteghini C, Paielli N, Licitra L, Pilotti S, Perrone F (2016). Prognostic and predictive value of EGFR in head and neck squamous cell carcinoma. Oncotarget.

[3] Vermorken JB, Trigo J, Hitt R, Koralewski P, Diaz-Rubio E, Rolland F, Knecht R, Amellal N, Schueler A, Baselga J (2007). Open-label, uncontrolled, multicenter phase II study to evaluate the efficacy and toxicity of cetuximab as a single agent in patients with recurrent and/or metastatic squamous cell carcinoma of the head and neck who failed to respond to platinum-based therapy. J Clin Oncol.

[4] Seiwert TY, Fayette J, Cupissol D, Del Campo JM, Clement PM, Hitt R, Degardin M, Zhang W, Blackman A, Ehrnrooth E, Cohen EE (2014). A randomized, phase II study of afatinib versus cetuximab in metastatic or recurrent squamous cell carcinoma of the head and neck. Ann Oncol.

[5] Egloff AM, Grandis JR (2009). Improving response rates to EGFR-targeted therapies for head and neck squamous cell carcinoma: candidate predictive biomarkers and combination treatment with Src inhibitors. J Oncol.

[6] Xu MJ, Johnson DE, Grandis JR (2017). EGFR-targeted therapies in the post-genomic era. Cancer Metastasis Rev.

[7] Marshall ME, Hinz TK, Kono SA, Singleton KR, Bichon B, Ware KE, Marek L, Frederick BA, Raben D, Heasley LE (2011). Fibroblast growth factor receptors are components of autocrine signaling networks in head and neck squamous cell carcinoma cells. Clin Cancer Res.

[8] Singleton KR, Kim J, Hinz TK, Marek LA, Casas-Selves M, Hatheway C, Tan AC, DeGregori J, Heasley LE (2013). A receptor tyrosine kinase network composed of fibroblast growth factor receptors, epidermal growth factor receptor, v-erb-b2 erythroblastic leukemia viral oncogene homolog 2, and hepatocyte growth factor receptor drives growth and survival of head and neck squamous carcinoma cell lines. Mol Pharmacol.

[9] Hinz TK, Kleczko EK, Singleton KR, Calhoun J, Marek LA, Kim J, Tan AC, Heasley LE (2019). Functional RNAi screens define distinct protein kinase vulnerabilities in EGFR-dependent HNSCC cell lines. Mol Pharmacol.

[10] Price KA, Cohen EE (2015). Mechanisms of and therapeutic approaches for overcoming resistance to epidermal growth factor receptor (EGFR)-targeted therapy in squamous cell carcinoma of the head and neck (SCCHN). Oral Oncol.

[11] Vermorken JB, Mesia R, Rivera F, Remenar E, Kawecki A, Rottey S, Erfan J, Zabolotnyy D, Kienzer HR, Cupissol D (2008). Platinum-based chemotherapy plus cetuximab in head and neck cancer. N Engl J Med.

[12] Bonner JA, Harari PM, Giralt J, Azarnia N, Shin DM, Cohen RB, Jones CU, Sur R, Raben D, Jassem J (2006). Radiotherapy plus cetuximab for squamous-cell carcinoma of the head and neck. N Engl J Med.

[13] Chow LQ, Haddad R, Gupta S, Mahipal A, Mehra R, Tahara M, Berger R, Eder JP, Burtness B, Lee SH (2016). Antitumor activity of pembrolizumab in biomarker-unselected patients with recurrent and/or metastatic head and neck squamous cell carcinoma: results from the phase Ib KEYNOTE-012 expansion cohort. J Clin Oncol.

[14] Ferris RL, Blumenschein G, Fayette J, Guigay J, Colevas AD, Licitra L, Harrington K, Kasper S, Vokes EE, Even C (2016). Nivolumab for recurrent squamous-cell carcinoma of the head and neck. N Engl J Med.

[15] Cohen EEW, Bell RB, Bifulco CB, Burtness B, Gillison ML, Harrington KJ, Le QT, Lee NY, Leidner R, Lewis RL (2019). The Society for Immunotherapy of Cancer consensus statement on immunotherapy for the treatment of squamous cell carcinoma of the head and neck (HNSCC). J Immunother Cancer.

[16] Miyauchi S, Kim SS, Pang J, Gold KA, Gutkind JS, Califano JA, Mell LK, Cohen EEW, Sharabi AB (2019). Immune modulation of head and neck squamous cell carcinoma and the tumor microenvironment by conventional therapeutics. Clin Cancer Res.

[17] Jie HB, Srivastava RM, Argiris A, Bauman JE, Kane LP, Ferris RL (2017). Increased PD-1(+) and TIM-3(+) TILs during Cetuximab therapy inversely correlate with response in head and neck cancer patients. Cancer Immunol Res.

[18] Petroni G, Buque A, Zitvogel L, Kroemer G, Galluzzi L (2020). Immunomodulation by targeted anticancer agents. Cancer Cell.

[19] Kersh AE, Sasaki M, Cooper LA, Kissick HT, Pollack BP (2016). Understanding the impact of ErbB activating events and signal transduction on antigen processing and presentation: MHC expression as a model. Front Pharmacol.

[20] Pollack BP (2012). EGFR inhibitors, MHC expression and immune responses : Can EGFR inhibitors be used as immune response modifiers?. Oncoimmunology.

[21] Srivastava RM, Trivedi S, Concha-Benavente F, Hyun-Bae J, Wang L, Seethala RR, Branstetter BFT, Ferrone S, Ferris RL (2015). STAT1-induced HLA Class I upregulation enhances immunogenicity and clinical response to Anti-EGFR mAb Cetuximab therapy in HNC patients. Cancer Immunol Res.

[22] Song C, Piva M, Sun L, Hong A, Moriceau G, Kong X, Zhang H, Lomeli S, Qian J, Yu CC (2017). Recurrent tumor cell-intrinsic and -extrinsic alterations during MAPKi-induced melanoma regression and early adaptation. Cancer Discov.

[23] Brea EJ, Oh CY, Manchado E, Budhu S, Gejman RS, Mo G, Mondello P, Han JE, Jarvis CA, Ulmert D (2016). Kinase regulation of human MHC class I molecule expression on cancer cells. Cancer Immunol Res.

[24] Lulli D, Carbone ML, Pastore S (2016). Epidermal growth factor receptor inhibitors trigger a type I interferon response in human skin. Oncotarget.

[25] Kersh AE, Ng S, Chang YM, Sasaki M, Thomas SN, Kissick HT, Lesinski GB, Kudchadkar RR, Waller EK, Pollack BP (2018). Targeted therapies: immunologic effects and potential applications outside of cancer. J Clin Pharmacol.

[26] Gurule NJ, Heasley LE (2018). Linking tyrosine kinase inhibitor-mediated inflammation with normal epithelial cell homeostasis and tumor therapeutic responses. Cancer Drug Resist.

[27] Sisler DJ, Heasley LE (2019). Therapeutic opportunity in innate immune response induction by oncogene-targeted drugs. Future Med Chem.

[28] Lichtenberger BM, Gerber PA, Holcmann M, Buhren BA, Amberg N, Smolle V, Schrumpf H, Boelke E, Ansari P, Mackenzie C (2013). Epidermal EGFR controls cutaneous host defense and prevents inflammation. Sci Transl Med.

[29] Mascia F, Lam G, Keith C, Garber C, Steinberg SM, Kohn E, Yuspa SH (2013). Genetic ablation of epidermal EGFR reveals the dynamic origin of adverse effects of anti-EGFR therapy. Sci Transl Med.

[30] Bonner JA, Harari PM, Giralt J, Cohen RB, Jones CU, Sur RK, Raben D, Baselga J, Spencer SA, Zhu J (2010). Radiotherapy plus cetuximab for locoregionally advanced head and neck cancer: 5-year survival data from a phase 3 randomised trial, and relation between cetuximab-induced rash and survival. Lancet Oncol.

[31] McCoach CE, Blumenthal GM, Zhang L, Myers A, Tang S, Sridhara R, Keegan P, Pazdur R, Doebele RC, Kazandjian D (2017). Exploratory analysis of the association of depth of response and survival in patients with metastatic non-small-cell lung cancer treated with a targeted therapy or immunotherapy. Ann Oncol.

[32] Sunakawa Y, Ichikawa W, Tsuji A, Denda T, Segawa Y, Negoro Y, Shimada K, Kochi M, Nakamura M, Kotaka M (2017). Prognostic impact of primary tumor location on clinical outcomes of metastatic colorectal cancer treated with cetuximab plus oxaliplatin-based chemotherapy: a subgroup analysis of the JACCRO CC-05/06 Trials. Clin Colorectal Cancer.

[33] Tsuji A, Sunakawa Y, Ichikawa W, Nakamura M, Kochi M, Denda T, Yamaguchi T, Shimada K, Takagane A, Tani S (2016). Early tumor shrinkage and depth of response as predictors of favorable treatment outcomes in patients with metastatic colorectal cancer treated with FOLFOX Plus Cetuximab (JACCRO CC-05). Target Oncol.

[34] Burkholder B, Huang RY, Burgess R, Luo S, Jones VS, Zhang W, Lv ZQ, Gao CY, Wang BL, Zhang YM, Huang RP (2014). Tumor-induced perturbations of cytokines and immune cell networks. Biochim Biophys Acta.

[35] Landskron G, De la Fuente M, Thuwajit P, Thuwajit C, Hermoso MA (2014). Chronic inflammation and cytokines in the tumor microenvironment. J Immunol Res.

[36] Nagarsheth N, Wicha MS, Zou W (2017). Chemokines in the cancer microenvironment and their relevance in cancer immunotherapy. Nat Rev Immunol.

[37] Thomas GR, Chen Z, Oechsli MN, Hendler FJ, Van Waes C (1999). Decreased expression of CD80 is a marker for increased tumorigenicity in a new murine model of oral squamous-cell carcinoma. Int J Cancer.

[38] Robinson MD, McCarthy DJ, Smyth GK (2010). edgeR: a Bioconductor package for differential expression analysis of digital gene expression data. Bioinformatics.

[39] Ritchie ME, Phipson B, Wu D, Hu Y, Law CW, Shi W, Smyth GK (2015). limma powers differential expression analyses for RNA-sequencing and microarray studies. Nucleic Acids Res.

[40] Korotkevich G, Sukhov V, Sergushichev A (2019). Fast gene set enrichment analysis. BioRxiv.

[41] Liberzon A, Subramanian A, Pinchback R, Thorvaldsdottir H, Tamayo P, Mesirov JP (2011). Molecular signatures database (MSigDB) 3.0. Bioinformatics.

[42] Bauerle KT, Schweppe RE, Lund G, Kotnis G, Deep G, Agarwal R, Pozdeyev N, Wood WM, Haugen BR (2014). Nuclear factor κB-dependent regulation of angiogenesis, and metastasis in an in vivo model of thyroid cancer is associated with secreted interleukin-8. J Clin Endocrinol Metab.

[43] Kleczko EK, Kim J, Keysar SB, Heasley LR, Eagles JR, Simon M, Marshall ME, Singleton KR, Jimeno A, Tan AC, Heasley LE (2015). An inducible TGF-beta2-TGFbetaR pathway modulates the sensitivity of HNSCC cells to tyrosine kinase inhibitors targeting dominant receptor tyrosine kinases. PLoS ONE.

[44] Gasteiger G, D'Osualdo A, Schubert DA, Weber A, Bruscia EM, Hartl D (2017). Cellular innate immunity: an old game with new players. J Innate Immun.

[45] Commins SP, Borish L, Steinke JW (2010). Immunologic messenger molecules: cytokines, interferons, and chemokines. J Allergy Clin Immunol.

[46] Tokunaga R, Zhang W, Naseem M, Puccini A, Berger MD, Soni S, McSkane M, Baba H, Lenz HJ (2018). CXCL9, CXCL10, CXCL11/CXCR3 axis for immune activation-a target for novel cancer therapy. Cancer Treat Rev.

[47] Tsukamoto H, Fujieda K, Senju S, Ikeda T, Oshiumi H, Nishimura Y (2018). Immune-suppressive effects of interleukin-6 on T-cell-mediated anti-tumor immunity. Cancer Sci.

[48] Ivashkiv LB, Donlin LT (2014). Regulation of type I interferon responses. Nat Rev Immunol.

[49] Newton K, Dixit VM (2012). Signaling in innate immunity and inflammation. Cold Spring Harb Perspect Biol.

[50] Barber GN (2015). STING: infection, inflammation and cancer. Nat Rev Immunol.

[51] Li J, Srivastava RM, Ettyreddy A, Ferris RL (2015). Cetuximab ameliorates suppressive phenotypes of myeloid antigen presenting cells in head and neck cancer patients. J Immunother Cancer.

[52] Seliger B (2014). The link between MHC class I abnormalities of tumors, oncogenes, tumor suppressor genes, and transcription factors. J Immunotoxicol.

[53] Ma W, Concha-Benavente F, Santegoets S, Welters MJP, Ehsan I, Ferris RL, van der Burg SH (2018). EGFR signaling suppresses type 1 cytokine-induced T-cell attracting chemokine secretion in head and neck cancer. PLoS ONE.

[54] Kang SH, Keam B, Ahn YO, Park HR, Kim M, Kim TM, Kim DW, Heo DS (2019). Inhibition of MEK with trametinib enhances the efficacy of anti-PD-L1 inhibitor by regulating anti-tumor immunity in head and neck squamous cell carcinoma. Oncoimmunology.

[55] Kuske M, Westphal D, Wehner R, Schmitz M, Beissert S, Praetorius C, Meier F (2018). Immunomodulatory effects of BRAF and MEK inhibitors: Implications for Melanoma therapy. Pharmacol Res.

[56] Srivastava RM, Trivedi S, Concha-Benavente F, Gibson SP, Reeder C, Ferrone S, Ferris RL (2017). CD137 stimulation enhances cetuximab-induced natural killer: dendritic cell priming of antitumor T-Cell immunity in patients with head and neck cancer. Clin Cancer Res.

[57] Ruzsa A, Sen M, Evans M, Lee LW, Hideghety K, Rottey S, Klimak P, Holeckova P, Fayette J, Csoszi T (2014). Phase 2, open-label, 1:1 randomized controlled trial exploring the efficacy of EMD 1201081 in combination with cetuximab in second-line cetuximab-naïve patients with recurrent or metastatic squamous cell carcinoma of the head and neck (R/M SCCHN). Invest New Drugs.

[58] Dietsch GN, Lu H, Yang Y, Morishima C, Chow LQ, Disis ML, Hershberg RM (2016). Coordinated activation of toll-like Receptor8 (TLR8) and NLRP3 by the TLR8 Agonist, VTX-2337, ignites tumoricidal natural killer cell activity. PLoS ONE.

[59] Vilgelm AE, Richmond A (2019). Chemokines modulate immune surveillance in tumorigenesis, metastasis, and response to immunotherapy. Front Immunol.

[60] Nazari A, Ahmadi Z, Hassanshahi G, Abbasifard M, Taghipour Z, Falahati-Pour SK, Khorramdelazad H (2020). Effective treatments for bladder cancer affecting CXCL9/CXCL10/CXCL11/CXCR3 axis: a review. Oman Med J.

