# Supplementary material for: Role of epidermal growth factor receptor inhibitor-induced interferon pathway signaling in the head and neck squamous cell carcinoma therapeutic response
Source: J Transl Med. 2021 Jan 23;19:43. doi: 10.1186/s12967-021-02706-8 (PMC7825244; doi:10.1186/s12967-021-02706-8)
Supplement: Supplementary file 1 — Additional file 1: Figure S1. Kinetics of gefitinib-stimulated interferon response gene induction in UMSCC8 and UMSCC25 cells. Total RNA was purified from UMSCC8 and UMSCC25 HNSCC cells treated for 2 hrs to 7 days with DMSO or gefitinib (300 nM) and submitted to RT-QPCR analyses for A, IFIT1, MX2, STAT1 and STAT2 with roles in innate immunity and B, the indicated chemokines and cytokines. The mRNA expression levels were normalized to GAPDH mRNA levels and presented as Relative Expression. The data points represent single determinations at six distinct time points per treatment. Figure S2. Multiplex analysis of chemokine and cytokine induction in HNSCC cell lines in response to the pan-ERBB inhibitor, AZD8931. A, The indicated human HNSCC cell lines were treated for 3 days with DMSO or 100 nM AZD8931 and conditioned media was analyzed in duplicate using a multiplexed Luminex assay for distinct secreted factors (see Materials and Methods). Expression levels for the analytes were normalized to the maximum measurement within the three cell lines and the data are presented as a heat map. Samples that were above or below the detection limit of the assay are indicated in grey. B, B4B8 cells were treated for 3 days with DMSO or AZD8931 (100 nM) and media was collected for a Luminex multiplexed assay for murine chemokines and cytokines. The data are presented as fold-stimulation by AZD8931 relative to DMSO treated cells. Figure S3. Sensitivity of murine HNSCC cell lines to EGFR/ERBB inhibitors. The murine HNSCC cell lines B4B8, MOC1, MOC2 and LY2 were submitted to clonogenic growth assays with triplicate determinations at each concentration of A, AZD8931 or B, gefitinib. Figure S4. Innate immune gene regulation by trametinib, but not AZD8931 in murine MOC2 HNSCC cells. MOC2 cells were treated for 3 days with DMSO, AZD8931 (100 nM) or trametinib (30 nM). RNA was purified and submitted to RT-QPCR for the innate immune genes IFIT3, MX2, STAT1 and STAT2 and expression was normal [file 12967_2021_2706_MOESM1_ESM.docx]

**Additional file for**

**Role of epidermal growth factor receptor inhibitor-induced interferon pathway signaling in the head and neck squamous cell carcinoma therapeutic response**

Sean P. Korpela^1^, Trista K. Hinz^1^, Ayman Oweida^5^, Jihye Kim^2^, Jacob Calhoun^1^, Robert Ferris^7^, Raphael A. Nemenoff^2^, Sana D. Karam^3^, Eric T. Clambey^4^ and Lynn E. Heasley^1,6^

Departments of Craniofacial Biology^1^, Medicine^2^, and Radiation Oncology^3^, and Anesthesiology^4^, University of Colorado Anschutz Medical Campus, Aurora, Colorado

^5^Department of Nuclear Medicine and Radiobiology, Universite de Sherbrooke, Sherbrooke, Québec, Canada

^7^Departments of Otolaryngology and Immunology, University of Pittsburgh, Pittsburgh, Pennsylvania

^6^Eastern Colorado VA Healthcare System, Rocky Mountain Regional VA Medical Center, Aurora, Colorado

**Additional file 1 Figure S1. Kinetics of gefitinib-stimulated interferon response gene induction in UMSCC8 and UMSCC25 cells.** Total RNA was purified from UMSCC8 and UMSCC25 HNSCC cells treated for 2 hrs to 7 days with DMSO or gefitinib (300 nM) and submitted to RT-QPCR analyses for **A**, IFIT1, MX2, STAT1 and STAT2 with roles in innate immunity and **B**, the indicated chemokines and cytokines. The mRNA expression levels were normalized to GAPDH mRNA levels and presented as Relative Expression. The data points represent single determinations at six distinct time points per treatment.

**Additional file 1 Figure S2. Multiplex analysis of chemokine and cytokine induction in HNSCC cell lines in response to the pan-ERBB inhibitor, AZD8931.** **A**, The indicated human HNSCC cell lines were treated for 3 days with DMSO or 100 nM AZD8931 and conditioned media was analyzed in duplicate using a multiplexed Luminex assay for distinct secreted factors (see Materials and Methods). Expression levels for the analytes were normalized to the maximum measurement within the three cell lines and the data are presented as a heat map. Samples that were above or below the detection limit of the assay are indicated in grey. **B**, B4B8 cells were treated for 3 days with DMSO or AZD8931 (100 nM) and media was collected for a Luminex multiplexed assay for murine chemokines and cytokines. The data are presented as fold-stimulation by AZD8931 relative to DMSO treated cells.

**Additional file 1 Figure S3. Sensitivity of murine HNSCC cell lines to EGFR/ERBB inhibitors.** The murine HNSCC cell lines B4B8, MOC1, MOC2 and LY2 were submitted to clonogenic growth assays with triplicate determinations at each concentration of **A**, AZD8931 or **B**, gefitinib.

**Additional file 1 Figure S4**. **Innate immune gene regulation by trametinib, but not AZD8931 in murine MOC2 HNSCC cells.** MOC2 cells were treated for 3 days with DMSO, AZD8931 (100 nM) or trametinib (30 nM). RNA was purified and submitted to RT-QPCR for the innate immune genes IFIT3, MX2, STAT1 and STAT2 and expression was normalized to GAPDH mRNA levels. The data points represent single determinations at three distinct time points per treatment.

**Additional file 1 Figure S5. EGFR/ERBB inhibitor-induced IFN pathway activation is dependent on IKK/NFκB and JAK signaling in human and murine HNSCC cell lines.** **A**, UMSCC8 and UMSCC25 cells were treated for 3 days with DMSO or gefitinib (300 nM) alone or in combination with ruxolitinib (1 uM) or IKK16 (500 nM). RNA was purified and submitted to RT-QPCR for the indicated genes and normalized to GAPDH mRNA levels. The maximum expression level for each gene among the two cell lines was used to normalize the distinct genes to a value of 1 and the data were presented as a heat map. **B**, UMSCC8 and UMSCC25 cells were treated for 3 days with DMSO, AZD8931 (100 nM) or trametinib (10 nM) in the presence or absence of ruxolitinib or IKK16. Conditioned media was collected and submitted to ELISA for human CXCL10. The data are the mean and SD of three independent experiments and presented as pg CXCL10 per μg of cellular protein. **C**, B4B8 cells were treated for 3 days with DMSO, AZD8931 (100 nM) or gefitinib (300 nM) in the presence or absence of ruxolitinib (1 uM) or IKK16 (500 nM). Conditioned media was collected and submitted to ELISA for murine CXCL10. The data are the mean and SD of three independent experiments and presented as pg CXCL10 per μg of cellular protein. **D**, B4B8 cells were transfected with an NFκB-responsive firefly luciferase reporter plasmid and a thymidine kinase-driven renilla luciferase reporter to estimate transfection efficiency. Following a 24-hour incubation, the transfected cells were treated with DMSO or AZD8931 alone or in combination with IKK16 or ruxolitinib. The data are the mean and SD of 3 independent experiments, and presented as fold-stimulation relative to DMSO treated cells. **E**, B4B8 cells were transduced with a retroviral vector encoding a dominant-negative IκB construct or an empty vector as a control (see Materials and Methods) and selected for puromycin resistance. The resulting cultures were treated for 3 days with DMSO, AZD8931 (100 nM), gefitinib (300 nM) or trametinib (10 nM) and conditioned media was submitted to ELISA for murine CXCL10. The data are the mean and SD of three independent experiments.

**Additional file 1 Figure S6. EGFR/ERBB inhibition augments expression of antigen presentation genes in human and murine HSNCC cell lines *in vitro*. A,** The human HNSCC cell lines UMSCC8 and UMSCC25 were treated with the EGFR inhibitor gefitinib (300 nM) over a time course of 1 to 7 days. RNA was submitted to RT-QPCR analyses for the MHC class I (B2M) and II (HLA-DMA) genes. The mRNA expression levels were normalized to GAPDH mRNA levels and presented as Relative Expression. **B,** The murine HNSCC cell line B4B8 was treated with the pan-ERBB inhibitor AZD8931 (100 nM) over a time course of 1 to 3 days and RNA was submitted to sequencing and expression of multiple MHC class I and II genes are reported as CPM. **C,** B4B8 cells were treated *in vitro* with DMSO or AZD8931 (100 nM) for 3 days, stained with PE-labeled anti-mouse MHC Class I (H-2Kd, H-2Dd; Invitrogen Clone 34-1-2S) or APC-labeled anti-mouse MHC Class II (I-Ad; eBioscience Clone AMS-32.1) and submitted to flow cytometry analysis. The median intensity of the fluorophore is presented, and the data are the mean of 2 independent experiments.
